# Clinical Characteristics and the Risk Factors of Hepatic Injury in 221 Children With Infectious Mononucleosis

**DOI:** 10.3389/fped.2021.809005

**Published:** 2022-01-12

**Authors:** Chao Zhang, Shu Cui, Guoshun Mao, Guitao Li

**Affiliations:** ^1^Department of Pediatrics, Fuyang People's Hospital, Fuyang, China; ^2^Chaohu Hospital, Anhui Medical University, Hefei, China; ^3^School of Mental Health and Psychological Sciences, Anhui Medical University, Hefei, China

**Keywords:** Epstein-Barr virus, infectious mononucleosis, hepatic injury, correlative factors, splenomegaly, gender

## Abstract

**Background:** Infectious mononucleosis caused by Epstein-Barr Virus infection is a common acute infectious disease in children. About 40–80% of children with infectious mononucleosis have hepatic injury, and hepatic failure is one of the main causes of death in patients with fatal infectious mononucleosis. Identifying the demographics, presenting clinical characteristics and the risk factors of hepatic injury in infectious mononucleosis children are helpful to remind clinicians which patients are prone to have hepatic damage.

**Methods:** A descriptive, cross-sectional study with a 31-month retrospective review was performed on all infectious mononucleosis children hospitalized in the pediatric department of Fuyang People's Hospital. Demographic data, presenting features, radiology imaging, clinical and laboratory parameters, and clinical outcomes of infectious mononucleosis children were collected.

**Results:** Two-hundred twenty-one infectious mononucleosis inpatients were enrolled, and 43.9% (97/221) patients were considered to have a hepatic injury (defined as alanine amino transaminase > 40 U/L). Compared with patients without hepatic injury, hepatic injury patients were marked with a significantly higher percentage of hepatomegaly (31 vs. 49%), splenomegaly (58 vs. 81%) and palpebral edema (47 vs. 63%), higher age (3.05 ± 2.12 vs. 3.84 ± 2.44), hospitalization days (6.85 ± 2.64 vs. 8.08 ± 2.83), leukocyte (14.24 ± 5.32 vs. 18.53 ± 8.63), lymphocytes (9.48 ± 4.49 vs. 13.80 ± 7.47), the proportion of atypical lymphocytes (0.12 ± 0.07 vs. 0.15 ± 0.08) and aspartate aminotransferase (33.71 ± 10.94 vs. 107.82 ± 93.52). The results of correlation analysis and multiple linear regression analysis indicated that age (OR = 1.185; 95% CI = 1.035–1.357, *p* = 0.014), female (OR = 2.002, 95% CI: 0.261–0.955, *p* = 0.036) and splenomegaly (OR = 2.171, 95% CI: 1.018–4.628, *p* = 0.045) were independent risk factors of hepatic injury.

**Conclusions:** In this study, the hepatic injury was associated with gender, age, and splenomegaly, which improved our understanding of risk factors about hepatic injury among infectious mononucleosis children.

## Introduction

Infectious mononucleosis (IM) is an acute systemic disease caused by Epstein-Barr Virus (EBV) or, more rarely, cytomegalovirus (CMV). It typically manifested as a triad of fever, angina, and lymphadenectasis, as well as varying degrees of hepatic injury (1). EBV, a DNA herpes virus, can enter squamous epithelial cells and spread from person to person through oropharyngeal secretions (1). In addition, EBV infection can also have long-term adverse consequences, such as chronic active EBV infection (2), EBV infection-related cancers (including nasopharyngeal cancer, gastric cancer, Kaposi sarcoma, post-transplant lymphoproliferative disease, etc.) (3–5). The prevalence of EBV infection in the population can be as high as 90% (5).

Studies have confirmed that about 40–80% of children with EBV-IM will have abnormal hepatic function (5–7). Alanine aminotransaminase (ALT), which exists in the cytoplasm of the liver, is the most important criterion and basis for hepatic injury, and the ALT level is generally proportional to the severity of hepatic injury (8). Although most immunologically normal IM patients with hepatic injury are self-limiting and have a good prognosis, some patients still die of liver failure or require liver transplantation (5, 9, 10). Liver failure is one of the leading causes of death in patients with fatal IM (11). Identifying the demographics, presenting characteristics, and risk factors of hepatic injury in infectious mononucleosis children for improving the prognosis of these rare cases of severe hepatic injury.

This retrospective study of the clinical characteristics of children with EBV-IM, aimed to explore the risk factors of hepatic injury, which can provide a reference for clinical evaluation and treatment.

## Methods

### Participants

**Figure 1:**
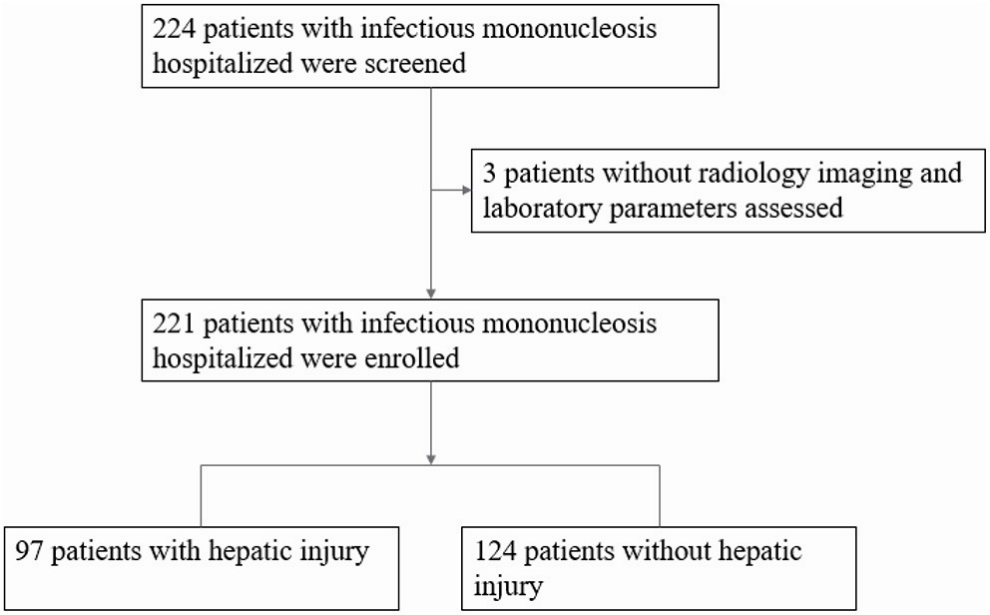
Flow chart of patient selection in this study.

Inpatients who were hospitalized from June 1, 2018 to January 1, 2021, were selected from the case system of Fuyang People's Hospital. All patients' parents have signed written informed consent at admission. This research scheme was approved by the Ethics Committee of Fuyang People's Hospital (No:2021-15). All procedures performed in this study involving human participants were following the Declaration of Helsinki. The flow chart of patient selection in this study is shown in Figure 1.

Inclusion criteria: (1) Age <14 years old when they were admitted, Han nationality; (2) Meet the IM diagnostic criteria. Diagnostic criteria: Meet any 3 of the clinical indicators and any 1 of the laboratory indicators. Clinical indicators: fever, pharyngeal tonsillitis, cervical lymphadenopathy, splenomegaly, hepatomegaly, eyelid edema. Laboratory indicators: (a) Anti-EBV-VCA-IgM and anti-EBV-VCA-IgG antibodies are positive, and anti-EBV-NA-IgG is negative; (b) Anti-EBV-VCA-IgM is negative, but anti-EBV-VCA-IgG antibodies are positive, and Low-affinity antibodies; (c) Double serum anti-EBV-VCA-IgG antibody titer increased by more than 4 times; (d) Peripheral blood atypical lymphocyte ratio ≥ 0.10 and (or) lymphocytosis ≥ 5.0 × 10^9^/L (12). (3) Patients' parents signed written informed consent on admission.

Exclusion criteria: (1) With other severe underlying systemic or organic diseases (especially hepatobiliary disease) may suffer from the elevation of ALT, such as fatty liver, gallstones et al. (2) Impaired renal function (serum creatinine > 160 μmol/L). (3) With other serious infectious diseases, such as Kawasaki disease, acute suppurative tonsillitis, suppurative lymphadenitis et al.

### Measure

Demographic data, presenting features, radiology imaging, clinical and laboratory parameters, and clinical outcomes were collected from the Electronic Case System of Fuyang People's Hospital. In this study, ALT ≥ 1 ULN (1 ULN: 40 U/L) was defined as combined hepatic injury according to previous literature (13, 14). Patients were divided into three groups according to hepatic injury defined by ALT level:(1) no hepatic injury (nHI) with ALT <1 ULN (<40 U/L), (2) mild hepatic injury (mHI) with ALT = 1–5 ULN and (3) severe hepatic injury (sHI) with ALT ≥ 5 ULN (13). Under ultrasound, the liver exceeded the costal margin by 2CM in children under 3 years of age, the liver exceeded the costal margin by 1CM in children between 3 and 7 years of age, and the liver exceeded the costal margin by 1CM in children over 7 years of age was defined as hepatomegaly. Splenomegaly was defined as the spleen exceeding the costal margin under ultrasound (Children need to keep calm breathing during ultrasound examination). Lymphadenectasis was defined as cervical lymph nodes with a short axis of >1 cm. Tonsillitis was defined as acute inflammation of the tonsils after infection, predominantly due to infection. Rash was defined as eruptions occurring after infection.

### Statistical Analysis

SPSS 22.0 software was used for statistical analysis. Shapiro-Wilk's normality test was conducted to examine normal distribution. Continuous variables were expressed as Mean ± SD. A chi-square test, Mann-Whitney U test, and *t*-test (Shapiro-Wilk test) were used to compare demographic and clinical data variables between the hepatic injury group and without hepatic injury group (defined using the cut-off scores of 1 ULN of ALT) (13, 14). Then Pearson correlation analysis was used to explore the relationship between the ALT and demographic and clinical data. Finally, multiple linear regression analysis (all factors are used including Gender, Age, CRP, Leukocyte, Lymphocyte, Proportion of atypical lymphocytes, Hepatomegaly, Splenomegaly, Palpebral edema) was used to explore the risk factors of the hepatic injury. *p*-values < 0.05 were deemed statistically significant. All statistical testing was two-tailed.

## Results

### General Demographic Data and Clinical Characteristics

Data from 224 children were collected in this study. Due to missing ALT, 221 children were included in the analysis, including 141 males (63.8%) and 80 females (36.2%). The mean age of onset was 3.40 ± 2.30 years, the mean hospitalization days was 7.38 ± 2.80 days, and 219 (94.6%) patients were associated with fever, and the mean duration of fever was 5.33 ± 3.20 days. The prevalence of nHI was 56.1% (124/221), mHI was 31.7% (70/221) and sHI was 12.2% (27/221), tonsillitis was 97.7% (216/221), palpebral edema was 53.8% (119/221), and the rash was 10.9% (24/221). The incidence of hepatomegaly was 39.4% (87/221), splenomegaly was 68.3% (151/221), and lymphadenectasis was 84.6% (187/221). Two hundred thirteen patients (96.4%) recovered and were discharged from the hospital, while 8 patients (3.6%) were not discharged or transferred. Abnormal indicators in the auxiliary examination include: In the peripheral blood of 221 children, ALT was 85.53 ± 128.60 U/L, Aspartate aminotransferase (AST) was 66.24 ± 72.40 U/L, C-reactive protein (CRP) was 11.99 ± 13.93 mg/L, Creatine kinase-myocardial band (CK-MB) was 26.34 ± 12.82 U /L, leukocyte count was 16.20 ± 7.25 × 10^9^/L, lymphocyte count was 11.41 ± 6.33 × 10^9^/L, the ratio of atypical lymphocytes was 0.13 ± 0.08. Serum EBV DNA was only measured in 114 children, which was 2.96 ± 1.01 log_10_ copies/mL.

### Clinical Characteristics of Children With Hepatic Injury

Table 1 summarizes the differences in demographic and clinical characteristic between patients without (*n* =124) and with hepatic injury (*n* = 97). No significant difference was observed between groups in regards to gender (χ^2^ = 3.77), fever duration (*t* = 0.25), CK-MB (*t* = −0.79) and Epstein - Barr virus DNA level (*t* = −0.64) and swollen lymph nodes (85 vs. 83%, χ^2^ = 0.16, df = 1), tonsillitis (99 vs. 96%, χ^2^ = 1.42, df = 1), rash (13 vs. 8%, χ^2^ = 1.22, df = 1) (all *P* > 0.05). However, significant differences were observed between groups in palpebral edema (47 vs. 63%, χ^2^ = 5.69, df = 1, *P* = 0.017), hepatomegaly (31 vs. 49%, χ^2^ = 7.42, df = 1, *P* = 0.006), splenomegaly (58 vs. 81%, χ^2^ = 13.75, df = 1, *P* < 0.001), age (3.05 ± 2.12 vs. 3.84 ± 2.44; *t* = −2.5, *p* = 0.013), hospitalization days (6.85 ± 2.64 vs. 8.08 ± 2.83; *t* = −3.35, *p* = 0.001), leukocyte (14.24 ± 5.32 vs. 18.53 ± 8.63; *t* = −4.12, *p* < 0.001), lymphocytes (9.48 ± 4.49 vs. 13.80 ± 7.47; *t* = −5.03, *p* < 0.001), the proportion of atypical lymphocytes (0.12 ± 0.07 vs. 0.15 ± 0.08; *t* = −2.75, *p* = 0.006), CRP (14.12 ± 16.56 vs. 9.26 ± 8.95; *t* = 2.785, *p* = 0.006), AST (33.71 ± 10.94 vs. 107.82 ± 93.52; *t* = −7.76, *p* < 0.001).

**Table 1:** General demographic data and clinical characteristics at admission.

**Variable**	**Total patients (*N* = 221)**	**Hepatic injury**		**t/Z/**χ^2^****	***p***
		**No (*n*=124)**	**Yes (*n*=97)**		
**Gender**				3.77	0.052
Female	80 (36%)	38 (31%)	42 (43%)		
Male	141 (64%)	86 (69%)	55 (57%)		
Age	3.40 ± 2.30	3.05 ± 2.12	3.84 ± 2.44	−2.50	0.013
Hospitalization days	7.38 ± 2.80	6.85 ± 2.64	8.08 ± 2.83	−3.35	0.001
Leukocyte	16.20 ± 7.25	14.24 ± 5.32	18.53 ± 8.63	−4.12	<0.001
Lymphocytes	11.41 ± 6.33	9.48 ± 4.49	13.80 ± 7.47	−5.03	<0.001
Proportion of Atypical lymphocytes	0.13 ± 0.08	0.12 ± 0.07	0.15 ± 0.08	−2.75	0.006
CPR	11.99 ± 13.93	14.12 ± 16.56	9.26 ± 8.95	2.785	0.006
ALT	85.53 ± 128.60	20.55 ± 9.11	169.69 ± 160.77	−9.03	<0.001
AST	66.24 ± 72.40	33.71 ± 10.94	107.82 ± 93.52	−7.76	<0.001
CK-MB	26.34 ± 12.82	25.74 ± 8.56	27.11 ± 16.79	−0.79	0.430
Epstein - Barr virus DNA level (*n* = 114)	2.96 ± 1.01	2.39 ± 8.13 (*n* = 61)	3.62 ± 1.21 *(n* = 53)	−0.64	0.52
**Lymphadenectasis**				0.16	0.686
No		18 (15%)	16 (17%)		
Yes	187 (85%)	106 (85%)	81 (83%)		
**Tonsillitis**				1.42	0.234
No		1 (0.8%)	4 (4%)		
Yes	216 (98%)	123 (99%)	93 (96%)		
**Rash**				1.22	0.270
No		108 (87%)	89 (92%)		
Yes	24 (11%)	16 (13%)	8 (8%)		
**Palpebral edema**				5.69	0.017
No		66 (53%)	36 (37%)		
Yes	119 (54%)	58 (47%)	61 (63%)		
**Hepatomegaly**				7.42	0.006
No		85 (69%)	49 (51%)		
Yes	87 (39%)	39 (31%)	48 (49%)		
**Splenomegaly**				13.75	<0.001
No		52 (42%)	18 (19%)		
Yes	151 (68%)	72 (58%)	79 (81%)		

*CRP, C-reactive protein; AST, Aspartate aminotransferase; ALT, Alanine aminotransaminase; CK-MB, Creatine kinase-myocardial band*.

### The Risk Factors of Hepatic Injury in IM Patients

Correlation analysis showed significant correlations between ALT score and the following parameters: age (*r* = 0.17, *p* < 0.05), leukocytes (*r* = 0.33, *p* < 0.01), lymphocytes (*r* = 0.40, *p* < 0.01), atypical lymphocytes (*r* = 0.17, *p* < 0.05), and AST (*r* = 0.81, *p* < 0.01). Because gender and age are confounding factors that affect liver function, a partial correlation analysis that controls for gender and age showed that there was no significant correlation between EBV-DNA level and ALT (*R* = 0.076, *P* = 0.424) (Table 2). Further, we used the regression to analyze the risk factors for hepatic injury in IM patients. The overall multivariate regression model (*F* = 54.69, *p* < 0.001) is statistically significant. The model could correctly classify 69.7% of the subjects. The sensitivity, specificity, positive predictive value, and negative predictive value were 61.9, 75.8, 66.7, and 71.8%, respectively.

**Table 2:** Correlation analysis between ALT and demographic and clinical parameters.

**Variables**	**Age**	**CRP**	**CK-MB**	**Leukocytes**	**Lymphocytes**	**Proportion of Atypical lymphocytes**	**ALT**	**AST**	**EBV DNA level^**a**^ (*n* = 114)**
ALT	0.17*	−0.13	−0.03	0.33**	0.40**	0.17*	1.00	0.81**	0.08

*p < 0.05, ** p < 0.01, two-tailed test; CRP, C-reactive protein; CK-MB, creatine kinase-myocardial band; AST, Aspartate aminotransferase; ALT, alanine aminotransaminase; ^a^partial correlation analysis that controls for gender and age.

In the multiple linear regression analysis for associations with hepatic injury in IM patients (Table 3), the female patients were 2 times more likely to have hepatic injury compared to male patients (OR = 2.002, 95% CI: 0.261–0.955, df = 1, *p* = 0.036). Patients with splenomegaly were 2.171 times more likely to have hepatic injury compared to those without splenomegaly (OR = 2.171, 95% CI: 1.018–4.628, df = 1, *p* = 0.045). Regression analysis confirmed the association between age and hepatic injury (OR = 1.185; 95% CI = 1.035–1.357, df = 1, *p* = 0.014).

**Table 3:** Predictors generated by Binary Logistic Regression with hepatic injury as dependent variables.

**Variable**	**B**	**S.E**.	**Wald**	**df**	**Sig**.	**Exp (B)**	**95%CI. for EXP (B)**	
							**Lower bound**	**Upper bound**
Gender	0.69	0.33	4.40	1	0.036	2.00	0.26	0.96
Age	0.17	0.07	6.06	1	0.014	1.19	1.04	1.36
CRP	−0.02	0.01	2.63	1	0.105	0.98	0.95	1.01
Leukocyte	−0.03	0.06	0.21	1	0.648	0.97	0.86	1.10
Lymphocyte	0.14	0.08	3.50	1	0.062	1.15	0.99	1.34
Proportion of Atypical lymphocytes	1.50	2.15	0.49	1	0.484	4.49	0.07	300.43
Hepatomegaly	0.34	0.34	1.00	1	0.317	1.41	0.72	2.74
Splenomegaly	0.78	0.39	4.03	1	0.045	2.17	1.02	4.63
Palpebral edema	0.37	0.32	1.29	1	0.256	1.44	0.77	2.72

*CRP, C-reactive protein*.

### Gender Differences of Hepatic Injury in IM Children

There were no significant differences in demographic data, presenting features, radiology imaging, clinical and laboratory parameters between female and male IM patients except hospitalization days (8.14 ± 3.76 vs. 6.96 ± 1.94, *t* =2.61, *p* = 0.01). Notably, the level of ALT (101.51 ± 150.25 vs. 76.47 ± 114.10, *t* =1.39, *p* = 0.17), AST (79.66 ± 88.99 vs. 58.62 ± 60.06, *t* =1.89, *P* = 0.06) and hepatic injury rate in females (53 vs. 39%, χ^2^ = 1.39, *p* = 0.05) were slightly higher than that in males (Table 4).

**Table 4:** Gender differences of hepatic injury in IM children.

	**Gender**		**t/Z/**χ^2^****	***p***
	**Female (*n* = 80)**	**Male (*n* = 141)**		
Age	3.11 ± 1.89	3.57 ± 2.49	−1.55	0.12
Hospitalization days	8.14 ± 3.76	6.96 ± 1.94	2.61	0.01
Duration of fever	5.23 ± 3.47	5.38 ± 3.05	−0.35	0.73
Leukocytes, 10^9^/L	15.59 ± 6.65	16.55 ± 7.57	−0.94	0.35
Lymphocytes, 10^9^/L	11.28 ± 6.01	11.48 ± 6.52	−0.22	0.83
Proportion of Atypical lymphocytes	0.13 ± 0.08	0.13 ± 0.08	0.28	0.78
CPR	10.54 ± 10.73	12.80 ± 15.43	−1.16	0.25
AST	79.66 ± 88.99	58.62 ± 60.06	1.89	0.06
ALT	101.51 ± 150.25	76.47 ± 114.10	1.39	0.17
CK-MB	27.62 ± 17.75	25.62 ± 8.90	1.12	0.27
Epstein - Barr virus DNA level	3.87 ± 1.26 (*n* = 48)	2.30 ± 7.93 (*n* = 66)	0.82	0.42
**Hepatic injury**			3.77	0.05
No	38 (47%)	86 (61%)		
Yes	42 (53%)	55 (39%)		
**Lymphadenectasis**			0.43	0.51
No	14 (18%)	20 (14%)		
Yes	66 (82%)	121 (86%)		
**Tonsillitis**			0.42	0.52
No	3 (4%)	2 (1%)		
Yes	77 (96%)	139 (99%)		
**Rash**			0.58	0.45
No	73 (91%)	124 (88%)		
Yes	7 (9%)	17 (12%)		
**Palpebral edema**			3.78	0.05
No	30 (37%)	72 (51%)		
Yes	50 (63%)	69 (49%)		
**Hepatomegaly**			0.02	0.89
No	49 (61%)	85 (60%)		
Yes	31 (39%)	56 (40%)		
**Splenomegaly**			0.25	0.62
No	27 (34%)	43 (30%)		
Yes	53 (66%)	98 (70%)		

*CRP, C-reactive protein; AST, Aspartate aminotransferase; ALT, Alanine aminotransaminase; CK-MB, Creatine kinase-myocardial band*.

## Discussion

In this study, we analyzed factors related to hepatic injury in Chinese IM children, hepatic injury was associated with gender, age, and splenomegaly, which confirm our understanding of risk factors about hepatic injury among infectious mononucleosis children. Our findings provide evidence that if a child admitted to hospital with EBV-IM is female, or older, or accompanies splenomegaly, or all three, clinicians must be vigilant for liver damage or even life-threatening liver failure caused by EBV.

The symptoms of IM are not caused directly by a viral infection of B cells but by an immune response that is caused by cytokine secretion and invasion of tissues by large numbers of activated cytotoxic CD8 + T lymphocytes typically present in peripheral blood (15, 16). During EBV infection, the absolute number of peripheral blood CD8+ T cells increases 5–10-fold in IM patients compared with asymptomatic individuals (17, 18). Infiltrating EBV-infected CD8+ T-cells and proliferating NK cells lead to hepatic injury and cause hepatomegaly, splenomegaly, and other clinical features, palpebral edema is related to the compression of swollen lymph nodes in the neck (Caused by lymphocyte infiltration) that makes lymphatic drainage not smooth (1). The pathogenesis of hepatic injury caused by EBV infection is now inclined to be regarded as an indirect immune injury caused by EBV as an immune initiating factor. Cytotoxic T lymphocytes with CD8+ in the body after EBV infection are the most important effector cell population when the liver is invaded. EBV-infected CD8+ T lymphocytes can be selectively captured by the hepatic, and hepatic Kupfer cells express soluble molecules, including Fas ligand, IFN-r, etc., leading to liver immune damage. The pathological manifestations of liver damage caused by EBV infection are slight swelling, vacuolization, and necrosis of hepatic cells, accompanied by lymphocyte and monocyte infiltration (19–21). Liver biopsy results of IM patients showed significant infiltration of lymphocytes around the portal vein, significant degeneration and necrosis of individual liver cells, and significant Kupffer cell activation (22). Although hepatic injury is common in EBV infection, fatal liver failure is rare in IM patients, which is in line with our result. Most reported fatal EBV infections are associated with complicated immunodeficiency syndrome (23).

In the current study, compared with patients with normal ALT levels, IM patients with abnormal ALT levels had the characteristics of the higher proportion of palpebral edema, hepatomegaly, and splenomegaly, longer hospital stays, higher levels of leukocyte, lymphocytes, the proportion of atypical lymphocytes, and CRP, which is consistent with the results of previous studies (7, 24, 25). Previous studies have confirmed that about 40–80% of children with EBV-IM showed abnormal transferase levels, especially in ALT (5–7), which is in line with our results. ALT is the main indicator of hepatic function impairment, which distribute in the cytoplasm of liver cells. With the increase of the degree of hepatic injury, the permeability of the liver cell membrane increases, and ALT leakage from the cytoplasm of liver cells leads to the increase of ALT in serum (26, 27).

The results of this study indicate that the hepatic injury (ALT levels) of older children with IM is more severe than that of younger children with IM, which is consistent with previous studies (18, 28–30). There is no significant correlation between EBV-DNA level in peripheral blood and ALT in this study. The mechanism of liver cell damage is mainly mediated by immune mechanisms, not directly caused by EBV (15, 16). In the host infected with EBV, the immunoactivity is an important determinant of liver damage (18, 28), which explains why EBV infection has milder symptoms in infants and young children (this period when the body's cellular and humoral immune mechanisms are immature). In children, EBV is mostly asymptomatic, while 50 to 70 percent of adolescents and adults (ages 15–25) seem to have symptoms (29). Wang et al. reported the levels of atypical lymphocytes and liver enzymes were higher in young adults with infectious mononucleosis than in preschoolers (30). ALT levels increase with age for 2 reasons: 1) The ability of young children to synthesize IgG, IgA, and other immunoglobulins, as well as the antigen presentation ability of monocytes/macrophages increase with age (31). Stronger immune responses to EBV infection cause more damage to liver cells. 2) Humoral immunity and cellular immunity play an important role in the process of EBV infection and pathogenesis. After EBV primary infection, it stimulates B lymphocytes to secrete and produce immunoglobulins, triggering humoral immune responses, and the corresponding body is specific for EBV latency antigens Sexually toxic T lymphocytes proliferate in large numbers, followed by an amount of apoptosis. Only a few antigen-specific memory T cells are latent in the body and circulate with the blood for a long time. When EBV proliferates again and produces a large number of specific antigens, the memory T cells can quickly enter the activation State and proliferate in large quantities, resulting in a more rapid and effective immune response (32, 33).

In short, the clinical manifestations of acute EBV infection in infants and young children period is generally not typical but can appear diversified clinical symptoms in older children, because the infants' immune response-ability is weak, cannot produce sufficient immune response to viral infection, several kinds of immune cells and cytokines in view of the viruses may not be able to produce effective response, lymphocyte immune response is weak. In the study of EBV-hemophagocytic lymphohistiocytosis (EBV-HLH), the onset age of secondary HLH caused by infection was lower than that of HLH caused by other causes (34). Therefore, follow-up should be executed to infants to alert serious complications after being infected with EBV.

Our research shows that female IM patients are twice as likely to suffer from hepatic injury as males, which is in line with the previous study (27). Human studies have shown females have stronger humoral and immune responses to viral infections than males, which proved true in the case of HIV and SARS-CoV-2 infections (35–37). The exact mechanism may be related to estrogen and the X chromosome, which is not fully understood, but the fact that women suffer from autoimmune diseases at a much higher rate than men suggests the gender difference (35, 36). For example, in Primary Biliary Cirrhosis, the ratio of male to female is 1:9.

This study suggested that the risk of hepatic injury in patients with splenomegaly IM was 2.171 times higher than that without splenomegaly IM. The estimated frequency of splenomegaly in IM ranges from 41 to 61.5% and lasts for ~21 days (30, 38, 39). The positive rate of splenomegaly in this study (68%) was slightly higher, resulting from the splenomegaly in this study being derived from the B-ultrasound examination rather than the doctor's physical examination. Splenomegaly is often difficult to find in physical examinations, so ultrasound examination of splenomegaly often has a higher positive rate (38). Splenomegaly is caused by the infiltration of lymphocytes in the spleen. The increase in spleen volume is related to the number of B lymphocytes, CD8+ T cells, and NK cells infected by EBV (40). Therefore, this common pathological mechanism of splenomegaly and hepatic injury supports the relationship between splenomegaly and hepatic injury.

There were several limitations of the current study. Firstly, this was a cross-sectional study that could not demonstrate a causal relationship. Secondly, the cases in this study were confined to Anhui Province, and the results could not be representative of China. Therefore, we will verify these results in larger sample sizes and multi-center studies in the future. Thirdly, Although the liver length measured by ultrasound has a moderate correlation with the liver volume (41), hepatomegaly and splenomegaly are clinical entities, defined based on clinical examination. Ultrasound may be useful in determining the nature of the parenchymal presentation, but not reliable in defining neither hepatomegaly nor splenomegaly. To improve accuracy, we will define it based on clinical examination in future studies. Fourth, there is the lack of data about potential correlation of CD8+ lymphocytosis or biomarkers of lymphocyte activation and proliferation as soluble CD25 levels and other inflammatory cytokines. Last but not least, as a case retrospective study, certain important liver function indicators (such as total bilirubin, coagulation function) and immune function index cannot be obtained in the medical records, and cannot be remedied at this stage, unfortunately. In future studies, we will highlight the collection of more comprehensive index of hepatic injury and immune function to better describe the patient's hepatic injury and to establish a more accurate and reliable forecasting model.

## Conclusion

In conclusion, children with hepatic injury were marked with significantly higher age, leukocyte, lymphocytes, abnormal lymphocytes, and length of hospital stay, as well as the elevated percentage of hepatomegaly, splenomegaly, palpebral edema. After controlling for confounders, this study found among children with IM, females and older children had the more serious hepatic injury. Patients with splenomegaly were more than twice as likely to have a concomitant hepatic injury as those without splenomegaly. The current study improved our understanding of risk factors for hepatic injury among infectious mononucleosis children.

## Data Availability Statement

The original contributions presented in the study are included in the article/supplementary material, further inquiries can be directed to the corresponding author/s.

## Ethics Statement

The study was approved by the Ethics Board of Fuyang People's Hospital (No:2021-15). Written informed consent to participate in this study was provided by the participants' legal guardian/next of kin.

## Author Contributions

CZ: conception and design. CZ and GM: administrative support and provision of study materials or patients. CZ, SC, and GL: collection and assembly of data. SC and CZ: data analysis and interpretation. All authors: manuscript writing and final approval of manuscript.

## Funding

This work was supported by Fuyang People's Hospital.

## Conflict of Interest

The authors declare that the research was conducted in the absence of any commercial or financial relationships that could be construed as a potential conflict of interest.

## Publisher's Note

All claims expressed in this article are solely those of the authors and do not necessarily represent those of their affiliated organizations, or those of the publisher, the editors and the reviewers. Any product that may be evaluated in this article, or claim that may be made by its manufacturer, is not guaranteed or endorsed by the publisher.
